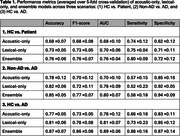# Decoding Dementia from Speech: Acoustic‐Lexical Integration for Detecting Alzheimer's Disease in Older Korean Adults

**DOI:** 10.1002/alz70856_101535

**Published:** 2025-12-25

**Authors:** Taehwan Kim, Sunghye Cho, Sung‐Woo Kim, Myungjin Ko, Mark Y Liberman, Min Seok Baek

**Affiliations:** ^1^ Silvia Health Inc., Seoul, Korea, Republic of (South); ^2^ Linguistic Data Consortium, University of Pennsylvania, Philadelphia, PA, USA; ^3^ Wonju Severance Christian Hospital, Yonsei University Wonju College of Medicine, Wonju, Korea, Republic of (South)

## Abstract

**Background:**

Early detection of mild cognitive impairment due to Alzheimer's disease (MCI d/t AD), as well as AD dementia (ADD), is critical for timely intervention. Speech analysis offers a non‐invasive way to detect subtle cognitive deficits. This study explores the utility of acoustic and lexical features in classifying older Korean adults across three clinical scenarios: (1) HC vs. AD (MCI d/t AD & ADD) for screening, (2) Non‐dementia (HC & MCI d/t AD) vs. ADD for detecting advanced pathology, and (3) HC vs. ADD for assessing the most divergent clinical states. We aim to demonstrate the feasibility of speech‐based methods for supporting more timely interventions.

**Method:**

We recruited 110 older Korean adults (HC=55, MCI d/t AD=29, ADD=26). Groups did not differ in gender (*p* = .372) or education (*p* = .278). However, the MCI d/t AD group was older (77.79±5.27) than the HC (72.51±6.38) and ADD (73.35±7.48) groups (*p* = .002), whereas there was no significant difference between HC and ADD. Cognitive measures (MMSE, CDR; both *p* <.001) differed significantly. All MCI d/t AD and ADD patients were beta‐amyloid positive in PET scans. Speech was collected via recording from neuropsychological tests and additional tasks (Korean phonemic/semantic fluency, vowel phonation, picture description). Acoustic and lexical features were extracted with openSMILE (emobase, 988‐dimensional) and a pretrained Korean RoBERTa model (768‐dimensional). Principal component analysis was applied to each feature set. Three classification models were built using (1) acoustic‐only, (2) lexical‐only, and (3) an ensemble of acoustic and lexical features. Each model was implemented through a multilayer perceptron and evaluated with 5‐fold cross‐validation.

**Result:**

In our experiments, ensemble models outperformed single‐feature‐based models (Table 1). For HC vs. AD, the ensemble model achieved 75.8% accuracy and 0.756 AUC; for Non‐dementia vs. ADD, 85.1% accuracy and 0.801 AUC; and for HC vs. ADD, 87.0% accuracy and 0.893 AUC. Combining acoustic and lexical features provided complementary information, reflecting vocal characteristics and language‐based deficits.

**Conclusion:**

These findings demonstrate that speech‐derived features can detect cognitive impairment in older Korean adults across multiple diagnostic scenarios, enabling earlier and more targeted interventions. Moreover, this non‐invasive approach may ease clinical workflows and broaden screening accessibility, particularly in resource‐limited settings.